# Effects of inflammatory cytokines on eradication and recurrence of Helicobacter pylori infection in children

**DOI:** 10.12669/pjms.38.7.5281

**Published:** 2022

**Authors:** Sisi Zhang, Yuanda Zhang, Songsong Chen, Yongnan Teng, Chen Li

**Affiliations:** 1 Sisi Zhang, Department of Gastroenterology, Baoding Children’s Hospital, Baoding, Hebei, 071000, P.R. China. Key Laborary of Clinical Research on Respiratory Digestive Disease, Hebei Baoding, 071000, China; 2 Yuanda Zhang, Department of Gastroenterology, Baoding Children’s Hospital, Baoding, Hebei, 071000, P.R. China. Key Laborary of Clinical Research on Respiratory Digestive Disease, Hebei Baoding, 071000, China; 3 Songsong Chen, Department of Gastroenterology, Baoding Children’s Hospital, Baoding, Hebei, 071000, P.R. China. Key Laborary of Clinical Research on Respiratory Digestive Disease, Hebei Baoding, 071000, China; 4 Yongnan Teng, Department of Gastroenterology, Baoding Children’s Hospital, Baoding, Hebei, 071000, P.R. China. Key Laborary of Clinical Research on Respiratory Digestive Disease, Hebei Baoding, 071000, China; 5 Chen Li, Department of Gastroenterology, Baoding Children’s Hospital, Baoding, Hebei, 071000, P.R. China. Key Laborary of Clinical Research on Respiratory Digestive Disease, Hebei Baoding, 071000, China

**Keywords:** Helicobacter pylori, IL-1β, IL-6, TNF- α, Eradication, Recurrence

## Abstract

**Objectives:**

To study the correlation of eradication and recurrence with IL-1 β, IL-6 and TNF-α levels in children with Helicobacter pylori (Hp) infection.

**Methods:**

Children confirmed with Hp infection (n = 153) in our hospital from January to June 2019 and successfully followed up for one year were selected as the study subjects, and 73 healthy children of the same period were selected as the control group. The levels of IL-1 β, IL-6 and TNF-α in children confirmed with Hp infection and healthy children were detected, respectively.

**Results:**

Results of triple therapy: 113 cases (73.9%) reexamined with negative 13C-urea breath test were included in Group-A; and 40 cases (26.1%) reexamined with positive 13C-urea breath test were included in Group-B. Follow up results: 23 cases (20.4%) positive in 13C-urea breath test in one year follow-up were included in Group-C; and 90 cases (79.6%) negative in 13C-urea breath test in one year follow-up were included in Group-D. Group-B showed significantly higher levels of IL-1β, IL-6 and TNF-α when compared with the Group-A and the control group, respectively (*P* < 0.05). Obviously higher levels of IL-1β, IL-6 and TNF-α were observed in Group-C and Group-D in comparison with the control group (*P* < 0.05). There was no significant difference in IL-1 β level between Group-C and Group-D (*P* > 0.05). IL-6 and TNF-α levels in Group-C were remarkably higher than those in Group-D (*P* < 0.05).

**Conclusions:**

Higher levels of IL-1β, IL-6 and TNF-α may affect the eradication effect of triple therapy in Children with Hp infection, and increased levels of IL-6 and TNF-α may affect the recurrence rate of Hp infection in children.

## INTRODUCTION

Helicobacter pylori (Hp) infection is chronic infectious disease, that globally affects half of the world’s population.^1^ In China, children are susceptible to Hp infection. This is mainly due to Chinese eating habits, which can be cross-infected by eating together. And the remission rate of Hp infection is relatively low without external therapeutic intervention.^2^ At present, the triple therapy of proton pump inhibitor (PPI) combined with two kinds of antibiotics is mainly used for eradication of Hp infection in children.^3^ However, the eradication rate of triple therapy showed a downward trend. Similarly, it has been reported that the eradication rate of the triple therapy against Hp infection has been declined, which is already below 80%.^4^ Therefore, it is very important to explore the reasons for the high recurrence rate of triple therapy and improve them accordingly. Moreover, some children with Hp infection would relapse after eradication treatment. children suffer from a high recurrence rate post eradication therapy.^5^ Current studies suggested that Hp infection could cause changes in the levels of interleukin-1β (IL-1β), interleukin-6 (IL-6), tumor necrosis factor-α (TNF-α) and other inflammatory cytokines.^6^ These pro-inflammatory cytokines are necessary and sufficient to induce villus damage and disrupt intestinal barrier function.^7^ To explore the correlation of eradication and recurrence with levels of IL-1β, IL-6 and TNF-α in children with Hp infection, the levels of IL-1β, IL-6 and TNF-α in children with Hp infection were detected and analyzed, and all patients were followed up for one year.

## METHODS

One hundred fifty three children with confirmed Hp infection in our hospital from January 2019 to June 2019 and successfully followed up for one year were selected as the study subjects, including 92 males and 61 females, with an average age of 9.1 years ± 2.4 years old. In addition, 73 healthy children of the same age groups were selected as the control group, including 44 males and 29 females, with an average age of 9.0 years ± 2.4 years old. Signed Informed written consent was obtained from the guardians of the children inducted in this study.

### Ethical Approval

The study was approved by the Institutional Ethics Committee of Baoding Children’s Hospital on June 8, 2020 (No.202035), and written informed consent was obtained from all participants

### Inclusion Criteria

- Children aged 4 to 16 years old;
- Patients with abdominal pain, nausea and other digestive system symptoms;
- Hp-positive patients diagnosed by endoscopic pathological staining and rapid urease test 8;
- Patients successfully followed up for 1 year.

### Exclusion Criteria:

- Patients with serious heart, lung, liver and kidney problems and endocrine, neurological or mental disorders.
- Patients with poor compliance and failure to complete the follow-up diagnosis.
- Patients with previous diagnosis of chronic diseases or acute infectious diseases in the past one month.
- Children with false positive HP infection.
- History of prior usage of PPI and antibiotics
- History of Upper Gi bleed regardless of the cause (causes alteration in the reading of rapid urease test)

### Triple Therapy used in this study

Omeprazole was given by one mg/kg. Daily, with a maximum dose being 20 mg/time, twice a day; Amoxicillin was given by 50 mg/kg. Daily with a maximum dose of one g/time, twice a day; Clarithromycin was given by 20 mg/kg. Daily with a maximum dose of 0.5 g/time, twice a day. Oral treatment was given for 14 days, drug withdrawal was carried out for four weeks at the end of treatment, and 13C-urea breath test was reexamined to evaluate the therapeutic effect. All children received the triple therapy, the 13C-urea breath test was reexamined after 4 weeks of withdrawal, and the 13C-urea breath test was reexamined half a year and one year after the 13C-urea breath test result turned into negative.

### Detection of IL-1β, IL-6 and TNF-α levels

Gastric mucosa samples were collected from all healthy children and Hp-infected children before treatment. Levels of IL-1β, IL-6 and TNF-α were detected with the double-sandwich microsphere capture method by the Baoding Key Laboratory of Clinical Research for Children’s Respiratory and Digestive Diseases, Baoding City, Hebei Province. The reagents were purchased from QuantoBio Biotechnology Co., Ltd., and the laboratory apparatus was Mindyay BriCyte E6 Flow cytometer. The detection shall be carried out by specially-assigned person with relevant qualifications.

### Gastric Mucosa Collection

Olympus V70 electronic gastroscope was used by qualified personnel to collect mucosal samples from five sites: two from the gastric antrum, two from the fundus of the stomach, and one from the gastric corner or small bend.

### Statistical Analysis

SPSS22.0 statistical software package was used for statistical analysis, measurement data were expressed as (*X̅*±*S*), while counting data were expressed in frequency and percentage. Elaborate and comparison between groups was analyzed by analysis of variance. *P* < 0.05 indicated that the difference was statistically significant.

## RESULTS

### Triple therapy results

among the 153 enrolled children, after drug withdrawal was carried out for four weeks at the end of treatment, 113 cases (73.9%) with negative results in reexamined 13C-urea breath test were included in Group-A, including 66 males and 47 females, with an average age of 9.0 ± 2.5 years. In addition, 40 cases (26.1%) with positive results in reexamined 13C-urea breath test were included in Group-B, including 26 males and 14 females, with an average age of 9.3 ± 1.9 years. There was no statistical significance in age and gender between Group-A, Group-B and control group (Table-I) (*P* > 0.05).

**Table I:** Eradication of triple therapy.

Grouping	Cases	Female (n)	Male (n)	Age (year)
Group-A	113 (73.9%)	66	47	9.0 ± 2.5
Group-B	40 (26.1%)	26	14	9.3 ± 1.9
Control group	73	44	29	9.0 ± 2.4

Among 113 children negative in reexamined 13C-urea breath test after the triple therapy, 23 cases (20.4%) who were positive in follow-up 13C-urea breath test were included in Group-C, including 13 males and 10 females, with an average age of 8.6 years ± 2.5 years old; 90 cases (79.6%) who were negative in follow-up 13C-urea breath test were included in Group-D, including 53 males and 37 females, with an average age of 9.1 years ± 2.5 years. There was no significant difference in age and gender among Group-C, Group-D and control group (Table-II) (*P* > 0.05).

**Table II:** Follow-up status after triple therapy.

Grouping	Cases	Female (n)	Male (n)	Age (year)
Group-C	23 (20.4%)	13	10	8.6 ± 2.5
Group-D	90 (79.6%)	53	37	9.1 ± 2.5

Comparisons of IL-1β, IL-6 and TNF-α levels among Group-A, Group-B and control group was made. The levels of IL-1β, IL-6 and TNF-α in Group-B were significantly higher when compared with Group-A and control group, respectively (*P*<0.05); specifically, Group-A showed significantly higher levels of IL-1β, IL-6 and TNF-α (*t* = 9.72; *t* = 4.60; *t* = 8.13; all *P* < 0.05). The results are shown in Table-III. These results suggest that increased levels of IL-1β, IL-6 and TNF-α may affect the eradication effect of triple therapy in children with Hp infection.

**Table III:** Comparisons of IL-1β, IL-6 and TNF-α levels among Group-A, Group-B and control group.

Grouping	Cases	IL-1β (pg/mL)	IL-6 (pg/mL)	TNF-α (pg/mL)
Group-A	113	12.2 ± 2.9	33.8 ± 10.1	14.2 ± 4.5
Group-B	40	19.8 ± 4.6	42.8 ± 11.9	25.3 ± 8.2
Control group	73	2.9 ± 1.0	5.5±1.7	3.3 ± 1.1
F value		471.59	315.07	290.69
P value		0.00	0.00	0.00

Comparisons of IL-1β, IL-6 and TNF-α levels among Group-C, Group-D and control group. The levels of IL-1β, IL-6 and TNF-α in Group-C and Group-D were obviously higher than those in the control group (*P* < 0.05). There was no significant difference in the IL-1β levels between Group-C and Group-D (*t* = 1.86, *P* > 0.05). IL-6 and TNF-α levels in Group-C were significantly higher than those in Group-D (*t* = 2. 09; t = 8.73, all *P* < 0.05). The results were shown in Table-IV. These results suggest that increased levels of IL-6 and TNF-α may affect the recurrence rate of Hp infected children.

**Table IV:** Comparisons of IL-1β, IL-6 and TNF-α levels among Group-C, Group-D and control group.

Grouping	Cases	IL-1β (pg/mL)	IL-6 (pg/mL)	TNF-α (pg/mL)
Group-C	23	20.5 ± 4.4	44.7 ± 8.7	26.8 ± 6.1
Group-D	90	18.8 ± 3.9	39.4 ± 10.1	15.1 ± 4.4
Control group	73	2.9 ± 1.0	5.5 ± 1.7	3.3 ± 1.1
F value		577.81	470.07	399.80
P value		0.00	0.00	0.00

## DISCUSSION

It has been demonstrated that the drug resistance for Helicobacter pylori (Hp) is on the rise.^9^ The eradication rates for PPI-based triple therapy having the combination of two kinds of antibiotics has a declining eradication rate in the treatment of Hp infection (less than 80%).^4^ The results of our study showed that the success rate of triple therapy in the eradication of Hp-infected children was 73.9% in this region, which is similar to one reported by Zhang M et al.^4^ It has been described that patient with Hp infection may relapse after taking triple therapy regimen, with the recurrence rate being 11.5% after one year.^10^ This recurrence rate was suggested to be related mainly to age, with younger children being associated with a higher risk of recurrence. In Another study, the recurrence rate within one year of therapy was 20% in children under the ages of nine years and 8% in children over 10 years of age respectively.^11^ Findings in our study showed that, after one year of *H.pylori* triple therapy regimen,, the recurrence rate in Hp-infected children between 4-16 years of age in our population in was 20.4%, indicating that the recurrence risk of Hp-infected children after eradication treatment in this region was higher Hence attention should be paid to the recurrence of Hp infection in children after eradication in clinical work.

Hp infection could stimulate the production of multiple inflammatory cytokines, such as IL-1β, IL-6 and TN-α. These inflammatory cytokines were suggested to be closely related to Hp infection related gastric inflammation.^12^ We found that children with Hp infection would have increased levels of IL-1β, IL-6 and TNF-α, which was consistent with the previous reports. Previous studies have reported that Hp could activate NOD-like receptor thermal protein domain associated protein 3 (NLRP3) inflammasome and induce the increase of IL-1β level through the ROS signaling pathway.^6^,^13^,^14^ Moreover, the genetic polymorphism of IL-1β was associated with chronic atrophic gastritis and gastric cancer,^15^ and relatively higher IL-1β level may be one of the reasons for the failure of eradication in children with Hp infection.^16^ Our results showed that, after triple therapy, the Hp-infected children with the failure of eradication had higher IL-1β levels than Hp-infected children with the success of eradication, which was consistent with previous reports, revealing that relatively high IL-1β level may affect the eradication effect of triple therapy.

IL-6 played an important role in gastrointestinal diseases.^17^ After the colonization of Hp in gastric mucosa, monocytes/macrophages and chronic inflammatory gastric tissues could be induced to produce IL-6.^18^ Our study highlighted that, after triple therapy, the level of IL-6 in children with positive 13C-urea breath test was higher than that in children with negative 13C-urea breath test, considering that the level of IL-6 may be a risk factor for the failure of Hp eradication. It was also noted that the initial IL-6 level in children with recurrence of Hp infection was higher than children without recurrence of Hp infection within one year after successful eradication, indicating that the increase of IL-6 level may affect the recurrence rate of Hp infection in children.

Currently it is believed that TNF-α plays an important role in the occurrence and development of gastric inflammation and peptic ulcer caused by Hp infection.^19^ There are elevated levels of TNF-α incases with Hp infection.^6^,^20^

### Limitations of the study

It includes single center study and small sample size.

## CONCLUSIONS

Results of our study showed that children with failed eradication of Hp infection by triple therapy and patients with relapsed Hp infection within one year after successful eradication of Hp infection had higher levels of TNF-α, suggesting that a high level of TNF-α may be associated with higher eradication failure rate and recurrence rate as for Hp infection. Therefore, the monitoring of inflammatory factors has positive guiding significance for the prevention of recurrence of HP infection in children.

### Authors’ Contributions:

**SZ &** **CL:** Designed this study and prepared this manuscript, are responsible for the accuracy and integrity of this study.

**YZ &** **SC:** Collected and analyzed clinical data.

**YT:** Significantly revised this manuscript.
